# Twitter Mentions Influence Academic Citation Count of Shoulder and Elbow Surgery Publications

**DOI:** 10.7759/cureus.21762

**Published:** 2022-01-31

**Authors:** Suleiman Sudah, Robert D Faccone, Matthew H Nasra, David Constantinescu, Mariano E Menendez, Allen Nicholson

**Affiliations:** 1 Orthopedic Surgery, Monmouth Medical Center, Long Branch, USA; 2 Research, Alabama College of Osteopathic Medicine, Dothan, USA; 3 Orthopaedic Surgery, Rutgers Robert Wood Johnson Medical School, New Brunswick, USA; 4 Orthopaedic Surgery, University of Miami, Miami, USA; 5 Orthopedic Surgery, Rush University Medical Center, Chicago, USA

**Keywords:** shoulder and elbow surgery, publications, research impact, citations, google scholar, social media, twitter

## Abstract

Background

Social media use among scholars and journals is growing and has augmented the academic impact of published articles in several areas of medicine. However, the influence of social media postings on academic citations of shoulder and elbow surgery publications is not known. In this study, we sought (1) to quantify the adoption of Twitter use for the dissemination of research publications by three prominent shoulder and elbow surgery journals and (2) to determine the correlation between Twitter mentions and academic citations in shoulder and elbow surgery publications.

Methodology

A total of 396 original research articles from three shoulder and elbow surgery journals (Journal of Shoulder and Elbow Surgery (JSES), Shoulder & Elbow, and JSES International) published in 2018 were assessed 34 to 45 months after print publication. For each article, the total number of Twitter mentions were obtained using Altmetric Bookmarklet and grouped into those tweeted by authors, an official outlet, or a third party. Article citation data was obtained using the Google Scholar search engine. Pearson correlation was used to determine the association between the number of Twitter mentions and citation count.

Results

Of all articles, 51% (202/396) had at least one Twitter mention. Of all Twitter mentions, 12.7% (367/2,879) occurred within the first week of online publication dates, while 51.5% (1,482/2,879) occurred between online and print publication dates. Articles mentioned on Twitter had 1.3-fold more Google Scholar citations (17.7 ± 15.2) than articles with no Twitter mentions (14.0 ± 15.7) (p = 0.017). The number of Twitter mentions had a weakly positive correlation with academic citation count (r = 0.25; p < 0.001). No significant difference in academic citation rates was found between articles tweeted by authors or official outlets when compared to articles tweeted by a third party only (p = 0.97 and p = 0.34, respectively).

Conclusions

Approximately half of shoulder and elbow surgery publications are shared on Twitter, with the majority of the activity occurring prior to their print publication date. The finding that tweeted articles have more academic citations within three years of release suggests that social media activity seems to amplify the academic impact of shoulder and elbow surgery publications.

## Introduction

The academic impact of publications has been traditionally measured by the number of referencing citations received in peer-reviewed journals and by the journal’s impact factor [1,2]. However, the broader social impact of a publication has gained importance [3,4] due to increasing social media participation within the academic community [5]. Alternative web-based metrics (Altmetrics) track the dissemination of research on various online platforms to provide a composite social attention score [6]. Unlike traditional bibliometrics, Altmetrics can quantify the social media impact a publication garners in real-time [7]. Because online social media platforms allow for the rapid promotion of research publications [8], it is plausible that social media engagement may influence citation rates and the overall academic impact of a research publication [9].

Twitter is the most commonly used social media platform for the spread of medical research [8], with more than 300 million active monthly users [10]. Over 80% of online mentions regarding musculoskeletal research occur on Twitter [8]. Twitter activity can serve as an early predictor of a publication’s citation number [11] and has been associated with an increase in subspecialty-specific literature citation [9,11-13]. However, because different subspecialties utilize social media and adopt new information-sharing techniques at different rates [14], it is critical that this evaluation be performed independently.

The influence of social media postings on academic citations of shoulder and elbow surgery publications is currently not known. In this study, we sought to quantify the adoption of Twitter use for the dissemination of original research publications by three prominent shoulder and elbow surgery journals and determine the correlation between Twitter mentions and academic citations in recent shoulder and elbow surgery publications.

## Materials and methods

Study eligibility and selection

The internet was used to perform a retrospective cross-sectional analysis of the shoulder and elbow surgery literature. Institutional review board approval was not required for this study. Electronic versions of all articles published in the Journal of Shoulder and Elbow Surgery (JSES), Shoulder & Elbow, and JSES International (previously called JSES Open Access in 2018) throughout the 2018 calendar year were analyzed. Original research articles and reviews were included. Editorials, opposing views, and case reports were excluded. The year 2018 was chosen to allow sufficient time for the articles to reach their citation potential, which has been shown to occur after three to five years of publication [15]. JSES, Shoulder & Elbow, and JSES International were selected because these journals are associated with the highest impact factors within the shoulder and elbow surgery literature [2,16,17].

Twitter mention and article citation data collection

Twitter mention and article citation data collection was performed in October 2021, approximately 34 to 45 months after the print publication of articles. The titles of included research articles were entered into the Google Scholar search engine (https://scholar.google.com/) and the “cited by” number provided with the resulting search query was obtained to determine the number of citations associated with each article. Google Scholar was chosen because it is publicly available and indexes all electronically available resources without limits on journal number, time period, or keywords [18]. Twitter mention data, including tweet date, content, and Twitter handle, were obtained from Altmetric Bookmarklet (https://www.altmetric.com/products/free-tools/bookmarklet/), which is a freely available internet browser extension that tracks social media attention around an article [19]. Publications self-tweeted by any author of an article were categorized as “author tweets,” whereas publications tweeted by the journal, publisher, or a national organization (American Shoulder and Elbow Surgeons (ASES)) were categorized as “official tweets.” All other tweets were considered “third-party” tweets.

Statistical analysis

A two-tailed t-test was used to compare the presence or absence of Twitter mentions, the presence or absence of author tweets, and the presence or absence of official tweets with the number of Google Scholar citations. Pearson correlation was used to determine the association between the number of Twitter mentions and citation count. The significance of all measurements was a two-sided P-value of <0.05.

## Results

A final sample of 396 original full-length scientific research articles, including 336 (85%) from JSES, 32 (8%) from Shoulder & Elbow, and 28 (7%) from JSES International were included for analysis (Table 1).

**Table 1:** Journal-specific Twitter mentions and Google Scholar citations. ^a^Values are presented as number. ^b^Values are presented as number (%). ^c^Values are presented as mean ± standard deviation. ^d^Data are presented as mean ± standard deviation and median (interquartile range).

Journal	Articles^a^	Tweeted articles^b^	Twitter mentions^c^	Official tweets^c^	Author tweets^c^	Google Scholar score^c^
Journal of Shoulder and Elbow Surgery	336	176 (52.4)	15.8 ± 53.0	0.5 ± 0.8	0.5 ± 1.1	18.7 ± 15.8
Shoulder & Elbow	32	12 (37.5)	5.7 ± 7.6	0.2 ± 0.6	0	10.3 ± 5.9
JSES International	28	14 (50)	2.6 ± 2.2	0.5 ± 0.7	0.2 ± 0.4	13.7 ± 9.6
Total^d^	396	202 (51)	14.3 ± 49.7 4 (10)	0.5 ± 0.8 1.3 (1)	0.5 ± 1.0 1 (1)	17.7 ± 15.2 14 (17.8)

Roughly 52.4% (176/336) of JSES articles had at least one Twitter mention compared to 37.5% for Shoulder & Elbow (12/32) and 50% (14/28) for JSES International. In total, 51% of articles had a minimum of one mention on Twitter (202/396), with an average of 14.3 ± 49.7 Twitter mentions per article (range 1-609). Of the 2,879 tweets analyzed, 2,671 (92.8%) were tweeted by a third party, 99 (3.4%) by an official source, and 91 (3.2%) by an author. There were an average of 0.5 ± 0.8 official tweets (range 1-5) and 0.5 ± 1.0 author tweets per article (range 1-7); most tweets were from third-party sources with an average of 13.2 ± 49.5 (range 1-609). The average Google Scholar score per tweeted article was 17.7 ± 15.2 (range 1-86).

**Figure 1:**
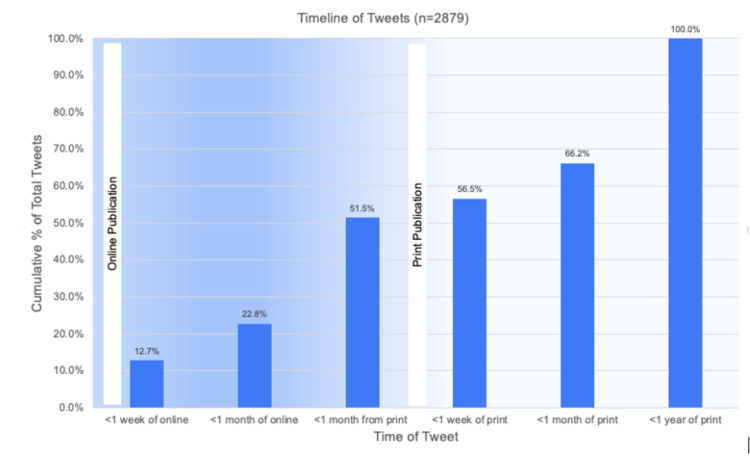
Timeline of cumulative tweets relative to electronic and print publication.

Upon examination of the timing of Twitter activity, we found that 51.5% (1,482/2,879) of Twitter mentions occurred prior to the print publication date when publications were released online. Specifically, the highest number of tweets (28.7%, 827/2,879) occurred within one month from the print publication. Roughly 13% (367/2,879) of Twitter mentions occurred within the first week of the electronic publication date. All Twitter activity ended within one year of print publication. A detailed breakdown of the Twitter mention timeline is depicted in Figure 1.

Articles mentioned on Twitter demonstrated a significant increase in Google Scholar citations (17.7 ± 15.2) compared to articles with no Twitter mentions (14.0 ± 15.7; 1.3-fold increase, p = 0.017) (Table 2).

**Table 2:** Average number of academic citations based on Twitter mentions. Data are presented as mean ± standard deviation and median (interquartile range).

	Tweeted (n = 202)	Not tweeted (n = 194)	P-value
Academic citations	17.7 ± 15.2 14 (17.8)	14.0 ± 15.7 10 (11)	0.017

Of all tweeted articles, 24.8% (50/202) were author tweets and 34.7% (70/202) were official tweets. There was no significant difference in Google Scholar citations among publications tweeted by their authors compared to non-author tweeted articles (17.8 ± 13 vs. 17.7. ± 15.9; p = 0.97) (Table 3).

**Table 3:** Average number of academic citations based on author self-tweets. Data are presented as mean ± standard deviation and median (interquartile range).

	Author tweet (n = 50)	Non-author tweet (n = 152)	P-value
Academic citations	17.8 ± 13 14.5 (15.8)	17.7 ± 15.9 14 (17.3)	0.97

There was no significant difference in the number of citations between publications tweeted by official outlets compared to articles not tweeted by official outlets (19 ± 17 vs. 17.1 ± 14.3; p = 0.43) (Table 4).

**Table 4:** Average number of academic citations based on official tweets. Data are presented as mean ± standard deviation and median (interquartile range).

	Official tweet (n = 70)	Non-official tweet (n = 132)	P-value
Academic citations	19 ± 17 14.5 (21)	17.1 ± 14.3 14 (15.3)	0.43

**Figure 2:**
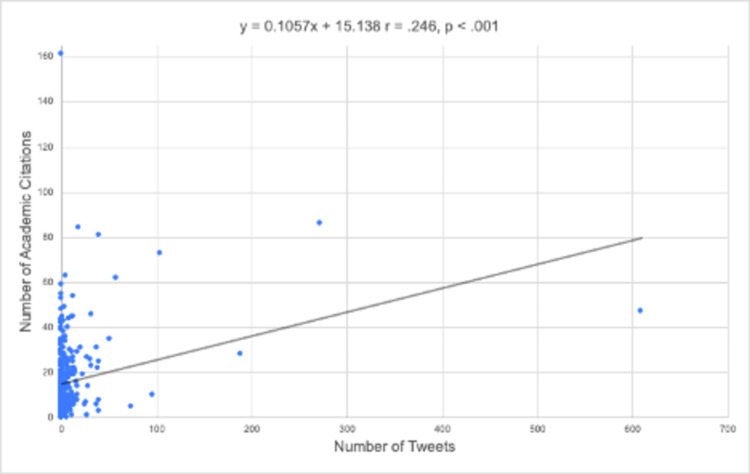
Scatterplot with linear regression line showing the number of academic citations versus the number of tweets.

Bivariate correlation analysis indicated that Twitter mention had a positive correlation with the citation rate of an article (r = 0.25; p < 0.001). A scatterplot with a linear regression line showing the number of academic citations versus the number of tweets is depicted in Figure 2.

## Discussion

This analysis of three reputable shoulder and elbow surgery journals demonstrated that shoulder and elbow surgery publications disseminated over Twitter were associated with a higher number of academic citations. Specifically, articles mentioned on Twitter averaged nearly four more academic citations than articles without Twitter mentions, representing a 1.3-fold increase (p = 0.017). While a cause-and-effect relationship is not afforded by this study, a positive correlation between the number of Twitter mentions and the number of academic citations was found (r = 0.25; p < 0.001). This suggests that the presence or absence of social media appears to play a role in the academic impact of shoulder and elbow surgery publications.

The results of our study are consistent with others of various medical specialties [9,11,12,20,21]. Twitter mentions have led to a 2.3-fold increase in Google Scholar citations for urology publications [15], a 1.6-fold increase for otolaryngology publications [9], and a 2.7-fold increase for vascular surgery publications [20]. The same holds true within the orthopedic literature. Zhang and Earp studied 835 articles published in Clinical Orthopaedics and Related Research, the Journal of the American Academy of Orthopaedic Surgeons, and the Journal of Bone and Joint Surgery from 2018 to 2019 [21]. The average number of Twitter mentions per article (1.1) was weakly correlated with the average number of Google Scholar citations per article (4.6). However, it is important to note that each of these journals encompasses articles pertaining to various subspecialties within orthopedic surgery. When analyzing three reputable hand surgery journals, Zhang et al. also found a weak positive correlation with the average number of Twitter mentions per article (1.3) and the average number of Google Scholar citations per article (3.9) [11]. Interestingly, our study demonstrates a greater number of Twitter mentions (14.3) and Google Scholar citations (17.7) per article within the shoulder and elbow surgery literature. These findings suggest that social media influences the academic impact of research articles differently across multiple areas of medicine and various subspecialties within the same surgical discipline.

Our study found variability in the percentage of articles with at least one Twitter mention among the three shoulder and elbow surgery journals studied. Roughly 52.4% (176/336) of JSES articles and 50% (14/28) of JSES International articles had at least one Twitter mention compared to 37.5% for Shoulder & Elbow (12/32). This may be because JSES is the most widely read shoulder and elbow surgery resource based upon it having the highest impact factor [2]. Perhaps this discrepancy in social media engagement represents an opportunity to leverage online platforms among different journals.

In recent years, several academic journals have adopted Twitter handles to augment their online presence [20]. Social media use among medical journals has been shown to increase journal page views [1,22], journal citations [23], and journal impact factors [24]. In our study, shoulder and elbow surgery publications tweeted by an official outlet resulted in a slight increase in the number of Google Scholar citations when compared to publications not tweeted by an official outlet (19 vs. 17.1). However, this finding was not significant and is likely confounded as official tweets also included those made by ASES and the journal publisher which was the same for each of the three journals studied (Elsevier). This data may suffer from a type II error because only 34.7% (70/202) of tweeted publications were tweeted by official outlets, with an average of 0.5 official tweets per article. Nonetheless, our findings indicate that shoulder and elbow surgery journals likely underutilize Twitter as a platform to disseminate their research publications.

A visual abstract is a graphic summary of a research publication that allows readers to quickly grasp what question a study addresses, the methodology used to examine this question, and the major findings [25]. Several journals have recently adopted the use of visual abstracts to promote and share research studies on social media [25]. The use of visual abstracts on Twitter was found to increase total engagement (defined as the total number of users who retweeted, replied to, liked, or expanded the tweet, followed the Twitter account, clicked on a link or hashtag, played embedded media, or clicked on the author’s profile photo or name) of nephrology publications nearly five-fold compared to citation-only tweets [25]. The JSES Family of Journals’ Twitter account (@JsesFamily) has recently implemented the use of visual abstracts as of October 2021. While our study is unlikely to account for the effects of visual abstracts on Twitter activity and academic citation rates within the shoulder and elbow surgery literature, we suspect that this change will amplify our findings in the foreseeable future.

Similarly, self-tweeted shoulder and elbow surgery publications demonstrated no significant difference in the number of Google Scholar citations when compared to non-self-tweeted publications (17.8 vs. 17.7). However, only 24.7% (50/202) of tweeted publications were tweeted by their authors, with an average of 1.8 author tweets per article. This comes as no surprise because a minority of shoulder and elbow surgeons utilize social media [26]. In a recent study, it was found that only 37% of 646 shoulder and elbow surgeons had a social media account (Twitter, 24.1%; Facebook, 23.1%; and Instagram, 9.4%) [26]. Conversely, Deshpande et al. demonstrated that self-tweeted otolaryngology articles had roughly eight more academic citations on Google Scholar when compared with non-self-tweeted articles (p < 0.01) [9]. Similarly, Hayon et al. demonstrated that self-tweeted urology articles were associated with roughly 12 and 15 more academic citations on Scopus and Google Scholar when compared to non-self-tweeted articles (p < 0.01 and p = 0.01, respectively) [12]. Perhaps with the more widespread adoption of social media within the shoulder and elbow surgery community, a greater number of authors will use Twitter to disseminate their research findings.

While academic citations have been a longstanding measure of research impact and productivity, they are associated with several drawbacks [11]. Citations take several years to accumulate and are therefore unable to gauge the academic impact of a publication in real-time [4]. In our study, we found that 51.5% of Twitter mentions occurred prior to the print publication date when publications were released online. Interestingly, 13% of Twitter mentions occurred within the first week of the electronic publication date, and all Twitter activity ended within one year of print publication. This Twitter engagement pattern indicates that social media momentum associated with electronic article release may correspond to the scholarly impact of shoulder and elbow surgery publications. Furthermore, the quantification of social media posts may be a more useful metric to assess research impact within the first year of print publication. Moreover, conventional academic citations fail to account for the impact of research on those who do not publish or have the ability to cite sources [21]. In our study, 92.8% of tweets were mentioned by a third party. Our findings suggest that Twitter may be increasingly used as a medium to discuss peer-reviewed shoulder and elbow surgery research among both scholars and the general public. Thus, social media mentions may further capture the societal impact of a research publication [27-29].

There are several limitations inherent to this study. First, the study only focuses on articles published in 2018. While this year was chosen to allow sufficient time for the maturation of conventional article citations [15], social media use rapidly expands each year [5]. Thus, it is possible that our findings may underestimate the strength of correlation between Twitter mentions and academic citations. Second, articles from only three shoulder and elbow surgery journals were studied. Therefore, our data do not account for all shoulder and elbow surgery research. However, a total of 396 original full-length articles were assessed, which is greater than what has been reported in similar studies [15,30]. We believe that this sample size provides an adequate representation of the shoulder and elbow literature. Importantly, however, an uneven distribution of publications was present among the three journals studied. It is likely that our findings are skewed toward JSES in particular. In addition, causality cannot be drawn from the results of this study. Therefore, it is unclear whether social media posting effectively disseminates shoulder and elbow surgery research, or whether high-impact research from reputable journals is more likely to garner social media attention. It is important to note that the presence of a few outliers within our dataset may have skewed our results in favor of tweeted articles, as demonstrated by large standard deviations. Finally, as is the case with all social media studies, the authors have no way of confirming the accuracy of the social media posts.

## Conclusions

Approximately half of shoulder and elbow surgery publications are shared on Twitter, with the majority of the activity occurring prior to their print publication date. The finding that tweeted articles have more academic citations within three years of release suggests that social media activity appears to amplify the academic impact of shoulder and elbow surgery publications. These findings suggest that Twitter activity can play an important role in the academic impact of shoulder and elbow surgery publications.
